# Probiotic Bacteria and their Supernatants Protect Enterocyte Cell Lines from Enteroinvasive *Escherichia coli* (EIEC) Invasion

**DOI:** 10.22088/acadpub.BUMS.6.3.183

**Published:** 2017-09-26

**Authors:** Zohreh Khodaii, Sayyed Mohammad Hossein Ghaderian, Mahboobeh Mehrabani Natanzi

**Affiliations:** 1 *Dietary supplements and Probiotic Research Center, Alborz University of Medical Sciences, Karaj, Iran.*; 2 *Urogenital Stem Cell Research Center, Shahid Beheshti University of Medical Sciences, Tehran, Iran.*

**Keywords:** Probiotics, enteroinvasive *Escherichia coli*, Caco-2 cells, T84 cells, adhesion

## Abstract

Probiotic microorganisms have attracted a growing interest for prevention and therapy of gastrointestinal disorders. Many probiotic strains have been shown to inhibit growth and metabolic activity of enteropathogenic bacteria as well as their adhesion and invasion to intestinal cells. In the present study, we evaluated the interference of bacteria-free supernatants (BFS) of cultures belonging to sixteen strains of *lactobacilli *and *bifidobacteria*, with invasion of enteroinvasive *Escherichia coli* (EIEC) strain, using human colonic adenocarcinoma cell lines, T84 and Caco2 cells.* To assess invasion of Caco-2 and T84 cells by EIEC, and measure the number of pathogens inside the enterocytes, the gentamicin protection assay was conducted. In addition, three different invasion inhibition assays were designed; namely co-incubation, pre-incubation and treatment with the BFS of probiotics. Data obtained and theoretical calculation showed that the most effective assay in the prevention of pathogen invasion was treatment with BFS. Besides, co-incubation assay was more valid than pre-incubation assay in invasion prevention.* *The obtained results suggest that probiotics may produce some metabolites that strongly prevent invasion of enteroinvasive *E.coli* into the small and large intestine. Also, probiotics are able to compete with or exclude pathogen* invasion.

*Escherichia coli* (*E. coli*) is part of the natural flora of human gut especially lower intestine, and one of the harmless flora of the gastrointestinal tract. It has also the pathogenic potential to cause significant diarrhea, and extra intestinal diseases, such as urinary tract, nervous system and respiratory system disorders (1).

The first step in the invasion process is adhesion of the bacteria to the epithelial cell. Therefore, the preservation of the intestinal flora can control the overgrowth of potentially pathogenic bacteria, and prevent related diseases. It is thought to be essential to prevent E. coli adhesion and invasion to preclude the pathogen from initiating an infectious process, and improve normal gut flora (2).

Recently, a growing public and scientific interest in probiotics bacteria has occurred to improve the health status of the host and avoid diseases caused by overgrowth of potentially pathogenic bacteria. Probiotics are live microorganisms which when administered in certain amounts, improve the gut microbial balance, and beneficially affect the host (3).

Several studies performed with different genera of probiotics bacteria, have shown the capacities of these bacteria to interfere with both growth and virulence properties of various pathogens. Probiotics exert antagonistic activity *in vivo* and *in vitro* against a range of gram positive and gram negative gastrointestinal pathogens (4). The mechanisms for the antibacterial activity of probiotics have been proposed to be antimicrobial metabolites, alteration of the intestinal evironmet by acid production, immune modulation of the host, competition for nutrients, adhesion to pathogens, and even blocking the adherence of the pathogens to the intestinal epithelial cells (5), which makes it possible for the strains to compete with specific pathogens for the receptor sites on host cells. Many recent studies have been conducted on anti-adherence activity of probiotics. In the present study, we examined the interference of sixteen strains of *lactobaccili* and *bifidobacteria*, as well as their bacteria free supernatants (BFS) with invasion of Enteroinvasive *E.coli*, using two cell lines derived from large and small intestines. 

## Materials and methods

**Bacterial strains, media and growth conditions**

Enteroinvasive *Escherichia coli* G24 (EIEC) was used as an invasive pathogen and non-invasive *E. coli* DH1 was used as a negative control. All *E.coli* strains were kindly donated by Dr. J. Fletcher (University of Bradford). The *E. coli* was grown aerobically at 37 C in Mueller-Hinton agar (Merck, Germany).

Probiotic strains were isolated from either pharmaceutical (5 strains), or dairy probiotic products (6 strains), and biochemical tests (gram stain, catalase, oxidase, carbohydrate fermentation patterns, effect of temperature on growth, acid tolerance, and resistance to bile salts) were used to identify them. Moreover, 16S rDNA gene sequencing was also used for bacterial identi-fication as previously reported (6). Besides, five culture types were obtained from National Collection of Industrial, Food and Marine Bacteria (NCIMB) Aberdeen, UK culture collection. Table 1 shows the list and source of strains.

**Table 1:** List and sources of tested probiotic and culture types.

**Probiotic strains**	**Source**
*Lactobacillus acidophilus *(*L. acidophilus *T)	NCIMB 701748
*Lactobacillus casei rhamnosus *(*L. rhamnosus *T)	NCIMB 8010
*Lactobacillus casei subspecies casei *(*L. casei *T)	NCIMB 11970
*Bifidobacterium bifidum *(*B. bifidum *T)	NCIMB 702715
*Bifidobacterium longum *(*B. longum *T)	NCIMB 702259
*L. acidophilus *(1C)	Advanced Acidophilus Plus Solgar Ltd.
*L. acidophilus *(2C)	Quest Digestive Aids: Quest Vitamines Ltd.
*L. acidophilus *(3C)	Multibionta: Seven Seas Ltd.
*L. acidophilus *(4C)	Health Aid acidophilus: Pharmadas Ltd.
*L. plantarum *(5C)	Children chewy Acidophilus : American Health Ltd.
*L. brevis *(1D)	Betta Buy Low fat fruit flavour yoghurt: Morrison`s
*L. sanfrancisco *(2D)	Low fat natural yoghurt: Morrison`s
*L. casei (Shirota) *(3D)	Yakult milk: Yakult
*Bifidobacterium spp. *(4D)	Activa: Danone France
*Bifidobacterium spp. *(5D)	Vitality yogurt Müller
*Bifidobacterium spp. *(6D)	Probiotic low fat yogurt: Tesco

**Probiotic culture condition**

All probiotic strains were cultured in the Man-Rogosa-and Sharpe (MRS) broth and agar (Merck, Germany), anaerobically at 37 ^o^C for 16-24 h and maintained at -20 C.

**Bacteria cell free supernatant preparation**

To prepare bacteria cell free supernatant of probiotics, 16 strains from the mentioned above sources, were cultivated in MRS broth for 18-24 h, under anaerobic condition. Bacteria-free superna-tant (BFS) was obtained by centrifugation at 3000 rpm in a Sigma 3-30k centrifuge for 20 min. To ensure the cell free status of BFS, supernatants were passed through a 0.4 μm pore size filter. 

**Cell lines culture**

The human colon adenocarcinoma cell line Caco-2 cells (CB No: 02D052) at seventh passage and the T84 colonic adenocarcinoma cell (The European Collection of Authenticated Cell Cultures, ECACC 88021101) at tenth passage were purchased from ECCAC and frozen in liquid nitrogen.

The Caco-2 human colon adenocarcinoma cells were routinely grown in a 95% air, 5% CO2 atmosphere at 37 C, in Dulbecco’s modified Eagle’s minimal essential medium (DMEM) from Sigma-Aldrich, supplemented with 10% (v/v) heat inactivated fetal bovine serum (FBS) (Gibco/Life technologies, Invitrogen), penicillin-streptomycin (100 IU/ml and 100 μg/ml, respectively).

The T84 culture medium composed of Ham's F12 + DMEM (1:1), 2 mM Glutamine and 10% FBS. T84 Cells were grown in a humidified atmosphere containing 5% CO_2 _at 37 C. Next, the cell lines were cultivated in 75 cm2 flasks, and were subsequently subcultured in the plates.

To conduct an assay, each cell line was seeded at a density of 2×10^5^ cells in 6-well tissue culture plates. The culture was refreshed every 2 days to form a monolayer culture, and was further cultivated for 7-10 days to reach confluent and differentiated cells. Then, monolayer cultures were incubated in an antibiotic-free medium for 24 h prior to treatment of the cells with bacteria, and cell invasion assay (7).

**Invasion assays**

To evaluate invasion of Caco-2 and T84 cells by EIEC, and measure the number of pathogens inside the enterocytes, the gentamicin protection assay was conducted with some modifications. Briefly, 3×10^7^ cfu/ml pathogen was added to each well. After 3 h incubation period, monolayers were washed 3 times with phosphate-buffered saline (PBS) and new media plus 50 μl of 1 mg/ml gentamicin (Sigma G1379) was added to each well to kill bacteria outside the enterocytes, but not those within the cells. Then, incubation was further continued for an hour. Afterwards, wells were washed 3 times with PBS and tissue culture cells were lysed by adding 2 ml 5% Na-deoxycholate (Sigma D 6750). Note that E. coli is resistant to sodium deoxycholate. Subsequently, a ten-fold dilutions of well contents were prepared and plated out on Mueller-Hinton agar. After overnight incubation at 37 ^o^C in aerobic atmosphere, the number of colony forming unit was determined, and the total internalized pathogen was calculated (8).

**EIEC invasion inhibition assay**

Three different invasion inhibition assays were designed, namely co-incubation, pre- incubation, and treatment with the bacteria free supernatant of probiotics to investigate differentiation, competition, exclusion, and probiotic BFS effect on EIEC (10). Besides, the intestinal cells and pathogenic bacteria without probiotic treatment were used as control.

In the co-incubation assay, either Caco2 cells or T84 cells were cultured, and then washed as described previously. Subsequently, 3×10^7^ cfu/ml of pathogen and 3×10^7 ^cfu/ml of probiotic bacteria were added simultaneously to each cell line, and incubated for 3 h under optimal conditions.

In the pre-incubation assay, probiotics were added to cell lines, and allowed to adhere to the cells for 3 h. Then, each well was washed with PBS and new medium plus 3×10^7^ cfu/ml pathogen was added into the wells and afterwards, incubation was further continued for 3 h.

In the supernatant treatment assay, the pathogen was treated with BFS from overnight culture of probiotic bacteria for 1 h. After treatment, the viability testing of the pathogen was performed. Then, the treated pathogen was added to the cell line at 3×10^7^ cfu/ml as previously described.

Eventually, the gentamicin protection assay was conducted and the number of internalized pathogens was determined by plating serial dilution on Muller Hinton agar. Each assay was performed 3 times in duplicate (9).

Data analysis

All data are expressed as means structural equation modeling (SEM). Statistical analysis was performed by repeated measures. Analysis of variance (ANOVA) and p-values < 0.05 were considered to be statistically significant.

## Results

**Invasion assay using T84 cells**

The cfu/ml number of internalized *E. coli* without any treatment of cell line or pathogen was 2×10^ 6^.

Co-incubation of probiotic strains was able to significantly reduce numbers of *E. coli *inside the T84 cells by at least two log cfu/ml. Among those tested, *L. acidophilus* showed less activity. Pre- incubation of all isolates were able to considerably reduce the number of internalized *E. coli *by at least one log _10_ cfu/ml. The only exception was *B. longum* which slightly decreased the number of invaded *E. coli*. Though, in supernatant treatment, the invasion ability of EIEC (G24) to cell line was decreased after incubation of the *E. coli* with BFS from an overnight broth culture of all test strains without *E. coli* viability decrease*.* In each case, the log number of *E. coli* inside the T84 cells after treatment with the BFS decreased about two log_10_ cfu/ml (Table 2). The most effective assay in the prevention of pathogen invasion was treatement with BFS.

**Table 2:** The effect of co-incubation, pre-incubation and treatment with bacterium free supernatant of test strains on *E. coli* G24 invasion into T84 cells.

**Probiotic strains**	**Type of assay**		
	**Supernatant treatment**	**Co-incubation**	**Pre-incubation**
*L. acidophilus *1C	(4.8±0.3)×10^ 2^	(3.5±0.45)×10^4^	(8±0.2)×10^4^
*L. acidophilus* 2C	(5±0.01)×10^ 2^	(3±0.2) ×10^4^	(5±0.1)×10^4^
*L. acidophilus* 3C	(4.75±0.25)×10^ 2^	(8.5±0.8)×10^4^	(5±0.2)×10^4^
*L. acidophilus* 4C	(4.8±0.2)×10^ 2^	(1.5±0.2)×10^4^	(1.3±0.1)×10^5^
*L. plantarum* 5C	(4.8±0.2)×10^ 2^	(1.8±0.2)×10^4^	(6±0.4)×10^4^
*L. brevis* 1D	(4.8±0.3)×10^ 2^	(1±0.3)×10^4^	(5±0.5)×10^ 2^
*L. sanfrancisco * 2D	(4.8±0.3)×10^ 2^	(2.5±0.3)×10^4^	(8.5±0.4)×10^4^
*L. casei Shirota* 3D	(4.8±0.5)×10^ 2^	(1.8±0.2)×10^4^	(5±0.3)×10^4^
*Bifidobacterium sp.* 4D	(4±0.3)×10^ 4^	(4.5±0.5)×10^3^	(3.5±0.4)×10^4^
*Bifidobacterium sp.* 5D	(5±0.2)×10^4^	(3±0.3)×10^3^	(6±0.6)×10^4^
*Bifidobacterium sp.* 6D	(1±0.1)×10^ 4^	(4±0.2)×10^4^	(1.3±0.2)×10^5^
*L. acidophilus *T	(5±0.35)×10^ 2^	(2±0.3)×10^4^	(6.5±0.2)×10^4^
*L. rhamnosus* T	(4.8±0.3)×10^ 2^	(1.5±0.4)×10^4^	(6.5±0.3)×10^4^
*L. casei* T	(5±0.4)×10^ 2^	(2±0.4)×10^4^	(5±0.2)×10^4^
*B. bifidum *T	(4.6±0.5×10^ 2^	(1.4±0.3)×10^4^	(1.5±0.1)×10^5^
*B. longum* T	(4.8±0.4)×10^ 2^	(2.4±0.3)×10^4^	(2.6±0.1)×10^5^

The numbers show the internalized *E. coli* colonies recovered at the end of each assay. The cfu/ml number of internalized *E. coli* without any treatment of cell line or pathogen was 2×10^ 6^.

The most effective strains in BFS, co-incubation, and pre-incubation were *L. acidophilus* 3C, *Bifidobacterium sp.* 5D, and *L. brevis* 1D, respectively. Moreover, co-incubation assay was more valid than pre-incubation assay in invasion prevention.

**Invasion assay using Caco2 cells**

The cfu/ml number of internalized *E. coli* without any treatment of cell line or pathogen was 2×10^ 6^.

Co-incubation of all isolates were able to decrease the number of *E. coli *inside the cells at least one log _10 _cfu/ml. The most effective strain was *B. longum T* that decreased the number of *E. coli* inside the Caco2 cells about two log _10 _cfu/ml.

Pre-incubation of probiotic strains was able to reduce the number of invaded *E. coli *to the Caco2 cells by at least one log _10 _cfu/ml in this assay format. Though, the less effective strain was *Bifidobacterium* 5D.

Invasion of EIEC G24 to Caco-2 cell was decreased after incubation of the *E. coli* with BFS from overnight cultures of all test strains without *E. coli* viability decrease (Table 3)*.* All isolates tested reduced the number of *E. coli* inside the Caco-2 cells by approximately three log _10_ cfu/ml.

**Table 3:** The effect of co-incubation, pre-incubation and treatment with bacterium free supernatant of test strains on *E. coli* G24 invasion of Caco-2 cells.

**Probiotic strains**	**Type of assay**		
	**Supernatant treatment**	**Co-incubation**	**Pre-incubation**
*L. acidophilus *1C	(5+0.1)×10^ 2^	(9+0.3)×10^ 4^	(4.5+0.2)×10^ 4^
*L. acidophilus* 2C	(5+0.2)×10^ 2^	(4.9+0.3)5×10^ 4^	(5.5+0.3)×10^ 4^
*L. acidophilus* 3C	(5+0.1)×10^ 2^	(3.5+0.2)×10^ 4^	(2+0.1)×10^ 4^
*L. acidophilus* 4C	(5+0.1)×10^ 2^	(4.8+0.2).×10^ 4^	(4.5+0.2)×10^ 4^
*L. plantarum* 5C	(5+0.2)×10^ 2^	(1+0.1)×10^ 4^	(1.5+0.1)×10^ 4^
*L. brevis* 1D	(5+0.1)×10^ 2^	(3+0.4).×10^ 4^	(5+0.3)×10^ 4^
*L. sanfrancisco * 2D	(5+0.1)×10^ 2^	(2.5+0.2)×10^ 4^	(2.5+0.1)×10^ 4^
*L. casei Shirota* 3D	(5+0.2)×10^ 2^	(2.5+01)×10^ 4^	(1.5+0.1)×10^ 4^
*Bifidobacterium sp.* 4D	(4+0.5)×10^ 4^	(4.5+0.5)×10^ 4^	(1.5+0.1)×10^ 5^
*Bifidobacterium sp.* 5D	(5+0.3)×10^ 4^	(4+0.2)×10^ 4^	(7.5+0.4)×10^ 5^
*Bifidobacterium sp.* 6D	(4+0.4)×10^ 4^	(5.2+0.2)×10^ 4^	(1.5+0.1)×10^ 5^
*L. acidophilus *T	(5+0.1)×10^ 2^	(4.2+0.2)×10^ 4^	(6+0.4)×10^ 5^
*L. rhamnosus* T	(5+0.1)×10^ 2^	(2+0.1)×10^ 4^	(2+0.2)×10^ 4^
*L. casei* T	(5+0.2)×10^ 2^	(1.5+0.1)×10^ 4^	(4.5+0.3)×10^ 4^
*B. bifidum *T	(5+0.3)×10^ 2^	(1.5+0.1)×10^ 4^	(3.5+0.3)×10^ 4^
*B. longum* T	(5+0.1)×10^ 2^	(1+0.1)×10^ 4^	(2.5+0.1)×10^ 4^

The above table shows the number of internalized *E. coli* colonies recovered at the end of each assay. The log _10 _cfu/ml of internalized *E. coli* without any treatment of cell line or pathogen was 2 ×10^ 6^.

The most effective assay in prevention of pathogen invasion was treatment with BFS where all strains showed the same effect except *Bifidobacteria*. In addition, the most effective strains were *L. plantarum* 5C, *B. longum* *T* in co-incubation and *L. plantarum* 5C, *L. casei Shirota* 3D in pre-incubation, respectively. Moreover, co-incubation assay was more effective than pre-incubation assay in invasion prevention.

## Discussion

After rotavirus, diarrheagenic Escherichia coli is the second most important of various etiological causes of diarrhea in infants and young children in the developing countries and among the travelers to these regions (9). Numerous studies have indicated that most of the probiotic strains have the capacity to inhibit growth and activity of enteropathogenic bacteria.

Considering that enterocyte invasion is an important virulence factor of *E. coli*, we examined the inhibitory effect of sixteen probiotic strains on *E. coli *invasion of Caco-2 and T84 cells, to find an effective probiotic bacteria or BFS. The results showed significant differences (P<0.05) between the invasion levels in the absence and presence of probiotic strains and their BFS.

Some recent research studies have documented the role of Lactobacillus in the prevention and treatment of diarrheal infections caused by Shigella, Salmonella and *E. coli *(10). Coconnier et al. have reported the ability of probiotic *Lactobacilli* and *Bifidobacteria* to inhibit cell association and invasion by pathogenic bacteria (11). Similarly, according to Altenhoefer et al. experimental data, Lactobacillus acidophilus strain LA1 inhibits cell invasion of Caco-2 cells by enteropathogenic E. coli, S. enterica serovar Typhimurium and Yersinia pseudotuberculosis *(*12*)*.

In our previous studies, we have shown the antibacterial activity of these sixteen probiotic strains (10). However, anti-invasive activity of probiotics and their BFS seems to be a secondary effect due to their bactericidal activity (3).

Another anti-invasion mechanism could be due to an anti-adhesive effect of probiotic bacteria or their BFS, through blocking of epithelial surface receptors by binding of probiotic strains and their metabolite to these receptors or by binding to the respective ligands of the invasive bacteria.

In the present study, treatment of the pathogen with BFS has been very effective to prevent invasion. It seems that the main mechanism for preventing pathogen invasion is not competitive inhibition of receptor adhesion, but through changes to the environment, cell barrier or gene expression by probiotic-induced metabolites.

These findings are supported by previous studies. For example Ramakrishna et al. showed that probiotics prevent pathogen adherence and invasion of the epithelium, partly by blocking adherence sites but also by upregulating gene expression of *MUC2* and of antimicrobial peptides. Production of short chain fatty acids is one of the effects of probiotic metabolites, which influence epithelial cell metabolism, turnover and apoptosis (13).

Resta-Lenert and Barrett showed that live Streptococcus thermophilus acidophilus protects the intestinal cells from the harmful effect of EIEC via several mechanisms that include, but are not restricted to, interference with pathogen adhesion and invasion. They proposed that probiotics may alter the cytoskeleton and tight junctions and limit ion transport dysfunction associated with EIEC infection of epithelial cells (2).

It should be noted that barrier and transport functions of the intestinal epithelium change mostly due to diverse digestive disorders, and are partly regulated by signal transduction events originating from the epidermal growth factor receptor (EGFR) and Lactobacillus (14). EIEC infection can prevent activation and increased degradation of the EGFR. Additionally, pretreatment of epithelial cells with probiotics might extend their ability to restore EGFR signaling after infection with EIEC (2).

It has been demonstrated that probiotics upregulate mucin gene expression in Caco-2 cells culture model. Mack et al. showed that part of the beneﬁcial effect of *L plantarum* and *L ramnosus* was mediated by induction of mucin genes in intestinal epithelial cells, and as expected for these probiotics, their BFS could interact with the level of mucin produced by Caco-2 and T84 cells and thus impair the adhesion of EIEC (15, 16).

In conclusion, in our study, we have explained that mechanisms involved in the pathogenic microbial invasion of eukaryotic cells are different depending upon the types of host tissues and microbial determinants. In future studies, we aim to investigate the mechanism of the anti-invasion activity of the most effective probiotic on Caco2 and T84 cells using molecular methods.
